# Persistent Amotivation After Cannabis‐Related Psychosis: Diagnostic Challenges and Clinical Course With Electroconvulsive Therapy: A Case Report

**DOI:** 10.1002/npr2.70181

**Published:** 2026-10-30

**Authors:** Yukina Kitano, Ryohei Takada, Minobu Ikehara, Kiyori Yamanaka, Yuka Sunami, Takashi Okada

**Affiliations:** ^1^ Department of Psychiatry Nara Medical University Kashihara Nara Japan

**Keywords:** apathy, cannabis use, cannabis‐induced psychotic disorder, electroconvulsive therapy, schizophrenia spectrum and other psychotic disorders

## Abstract

**Background:**

Cannabis use may cause psychotic symptoms, such as hallucinations and delusions, as well as persistent amotivation with reduced motivation, inactivity, and cognitive impairment. Although described as cannabis‐related amotivational syndrome, it is not recognized as an independent diagnostic entity and overlaps clinically with the negative symptoms of schizophrenia and mood disorders. We report a case of persistent amotivation following cannabis‐related psychosis that improved after electroconvulsive therapy (ECT).

**Case:**

A 19‐year‐old man developed auditory hallucinations, persecutory delusions, and agitation associated with cannabis use and was admitted to a psychiatric hospital. Antipsychotic treatment improved the psychotic symptoms, but marked amotivation and inactivity, accompanied by low performance on cognitive screening, and significantly impaired daily functioning. Depressed mood and appetite loss were not evident. Although negative symptoms of schizophrenia remained a differential diagnosis, the presentation was consistent with cannabis‐related amotivational syndrome. Amantadine produced limited improvement. Following ECT, the Apathy Scale score improved from 24 to 7 and the Mini‐Mental State Examination score increased from 19 to 26, with improvements in motivation, activity, and daily functioning.

**Discussion:**

This case demonstrates that clinically significant amotivation may persist after cannabis‐related psychotic symptoms improve. Persistently reduced motivation and inactivity should not be assumed to represent primary negative symptoms of schizophrenia; rather, clinicians should carefully consider mood symptoms and cannabis‐related amotivation in the differential diagnosis. This case also suggests that ECT may represent a treatment option for severe, persistent amotivation following cannabis‐related psychosis. However, because this was a single case, further validation through additional cases and long‐term follow‐up is required.

## Abstract 2

Clinically significant amotivation persisted after improvement of cannabis‐related psychotic symptoms. Following ECT, motivation, activity, and daily functioning improved markedly, suggesting that ECT may represent a potential treatment option for severe, persistent amotivation following cannabis‐related psychosis.

## Graphical abstract

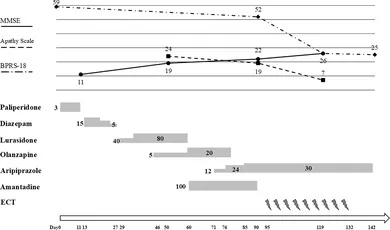

## 1 Introduction

Amotivational syndrome was first described by McGlothlin and West in 1968 as a constellation of chronic psychiatric symptoms observed in long‐term cannabis users [1]. It has been characterized by reduced initiative, affective flattening, difficulty sustaining concentration, reduced motivation, inactivity, and memory impairment [1, 2, 3]. However, it is not recognized as an independent diagnostic entity but rather as a historical and descriptive concept encompassing these symptoms [4].

Cannabis‐related hallucinations and delusions are classified in DSM‐5‐TR as a cannabis‐induced psychotic disorder [5]. In clinical settings, differentiating cannabis‐induced psychosis from schizophrenia spectrum disorders is often challenging. This distinction is important because substance‐induced psychosis may later transition to schizophrenia spectrum disorders, with cannabis‐induced psychosis carrying a relatively high risk [6, 7]. Therefore, when amotivation persists after cannabis use, careful differentiation from negative symptoms of schizophrenia is necessary.

In recent years, the relationship between cannabis use and motivation‐ or reward‐related symptoms has been re‐examined. Cannabis use may contribute to apathy, anhedonia, affective flattening, and reduced sensitivity to natural rewards [8]. Acute cannabis use may reduce effort expenditure for reward and has been associated with apathy. In contrast, studies evaluating persistent amotivational syndrome among non‐acute cannabis users have produced inconsistent findings [4]. Additionally, although the International Classification of Diseases, 11th Revision (ICD‐11) includes cannabis‐induced mood disorder, its boundary with symptoms characterized mainly by reduced motivation and activity remains unclear [9].

Persistent amotivation following cannabis use overlaps with negative symptoms and mood disorders, with no established standardized treatment. We report a 19‐year‐old man who developed persistent amotivation after improvement of cannabis‐related psychotic symptoms and showed improvement following electroconvulsive therapy (ECT).

## 2 Case

The patient was a 19‐year‐old man with no intellectual disability or developmental disorder. At age 14, he began using cannabis recreationally. Three years before admission, he began daily solitary use. Around the same period, he developed reduced motivation, impaired concentration, and auditory hallucinations, leading to absenteeism and job loss.

One year before admission, the patient jumped from the third floor of his home in response to auditory hallucinations. He was referred to our department but concealed both cannabis use and hallucinations. Retrospective interviews later revealed that these symptoms resolved spontaneously, approximately one month after cannabis cessation. However, he resumed cannabis use soon afterward and progressed to daily use.

In April of the admission year, he again attempted to jump in response to auditory hallucinations. At an outpatient visit on April 10, he reported hallucinations and delusional experiences, including statements like “I can see something like a haze” and “I feel the presence of people,” and was admitted. He disclosed cannabis use for the first time and reported continued use until shortly before admission. On admission, he showed reduced responsiveness and was unable to state his date of birth. His 18‐item Brief Psychiatric Rating Scale (BPRS‐18) score was 59 (Table 1), and his Global Assessment of Functioning (GAF) score was 20.

**TABLE 1:** The 18‐item Brief Psychiatric Rating Scale scores during hospitalization.

		Day 0	Day 90	Day 119	Day 142
1	Somatic concern	4	3	2	1
2	Anxiety	5	4	2	1
3	Emotional withdrawal	5	5	1	2
4	Conceptual disorganization	2	4	2	3
5	Guilt feelings	4	1	1	1
6	Tension	3	4	2	2
7	Mannerisms and posturing	1	2	2	2
8	Grandiosity	1	1	1	1
9	Depressive mood	4	4	1	1
10	Hostility	1	2	1	1
11	Suspiciousness	1	2	1	1
12	Hallucinatory behavior	6	2	1	1
13	Motor retardation	4	6	2	1
14	Uncooperativeness	3	2	1	2
15	Unusual thought content	2	2	2	2
16	Blunted affect	4	5	2	1
17	Excitement	2	1	1	1
18	Disorientation	7	2	1	1
	Total score	59	52	26	25

*Note:* Each item is rated from 1 (not present) to 7 (extremely severe). Each item is rated from 1 (not present) to 7 (extremely severe). Each item is rated from 1 (not present) to 7 (extremely severe).

Figure 1 illustrates the clinical course after admission. Blood tests, electroencephalography, brain magnetic resonance imaging, and cerebrospinal fluid examination revealed no significant abnormalities. On admission, urine drug screening with the Triage system did not detect any non‐cannabis substances. Paliperidone was initiated but discontinued because of elevated liver enzyme levels. Diazepam was started on day 13 because reduced spontaneity associated with stupor was considered possible. However, typical catatonic features such as negativism, posturing, stereotypy, echolalia, or echopraxia were absent. Although he remained inactive, dazed, and spent most of the day in bed, the hallucinations and delusions persisted. Diazepam was discontinued, and antipsychotic treatment resumed. Lurasidone and olanzapine produced insufficient improvement; however, after switching to aripiprazole on day 71 and increasing the dose to 30 mg on day 85, the visual hallucinations and delusions gradually improved.

**FIGURE 1:**
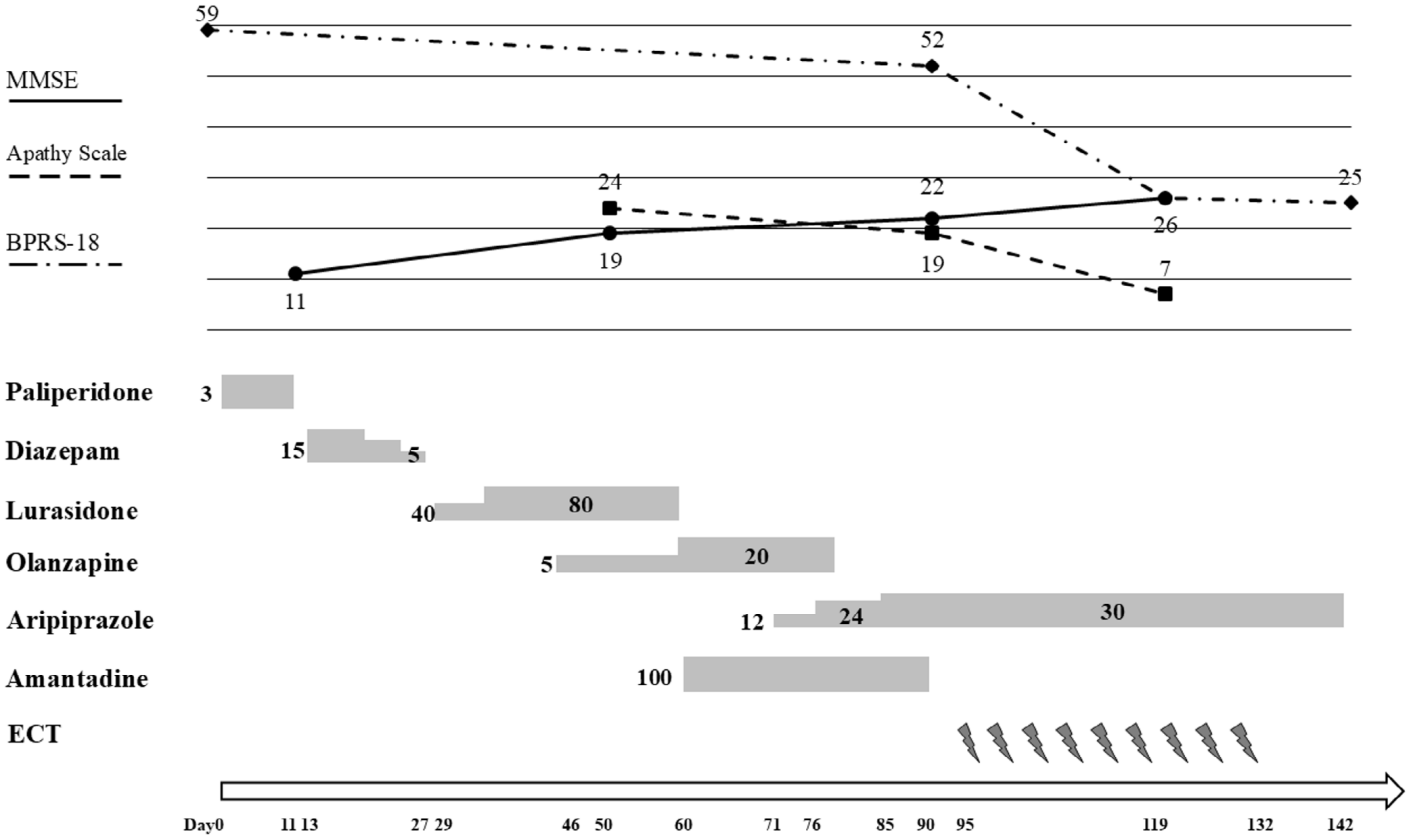
Clinical course after admission. The figure shows the temporal relationship between pharmacological treatments, ECT, and changes in Apathy Scale, MMSE, and BPRS‐18 scores. Numbers within medication bars indicate daily doses (mg/day). BPRS‐18, 18‐item Brief Psychiatric Rating Scale; ECT, electroconvulsive therapy; MMSE, Mini‐Mental State Examination.

Severe inactivity and reduced motivation persisted, with the patient spending most of the day in bed. On day 50, the Japanese version of the Apathy Scale (AS) [10, 11] score was 24 and the Mini‐Mental State Examination (MMSE) score was 19 (Table 2), indicating marked apathy with low performance on cognitive screening. Because amotivation and inactivity substantially impaired daily functioning, amantadine 100 mg/day was initiated on day 60 as a pharmacological trial targeting apathy. As psychotic symptoms persisted, he was closely monitored for potential worsening of hallucinations or delusions. No exacerbation was observed. By day 90, hallucinations and delusions had gradually improved with aripiprazole, but amotivation remained prominent. The MMSE score was 22, AS score was 19, and BPRS‐18 score was 52. Therefore, ECT was initiated.

**TABLE 2:** Apathy Scale scores during hospitalization.

		Day 50	Day 90	Day 119
1	Are you interested in learning new things?	3	3	1
2	Does anything interest you?	2	3	0
3	Are you concerned about your condition?	2	1	0
4	Do you put much effort into things?	2	2	0
5	Are you always looking for something to do?	1	0	0
6	Do you have plans and goals for the future?	2	1	1
7	Do you have motivation?	2	2	1
8	Do you have the energy for daily activities?	2	2	1
9	Does someone have to tell you what to do each day?	1	1	1
10	Are you indifferent to things?	1	1	1
11	Are you unconcerned with many things?	2	1	0
12	Do you need a push to get started on things?	2	1	1
13	Are you neither happy nor sad, just in between?	2	1	0
14	Would you consider yourself apathetic?	0	0	0
	Total	24	19	7

*Note:* For questions 1–8: not at all = 3 points; slightly = 2; some = 1; a lot = 0. For questions 9–14: not at all = 0; slightly = 1; some = 2; a lot = 3. A total score of 16 or higher indicates apathy.

Written informed consent was obtained from the patient and his family after a detailed explanation of ECT. ECT was initiated on day 95 and administered twice weekly, for a total of nine sessions over 5 weeks. Propofol was used for anesthesia, and suxamethonium for muscle relaxation. Bilateral stimulation was performed. Seizure adequacy was assessed based on successful seizure induction, generalization, Electroencephalogram (EEG) morphology, bilateral symmetry and synchrony, postictal suppression, and seizure duration. During the first session, an initial stimulus of 50.1 mC with a stimulus train duration of 5.6 s produced no detectable EEG or Electromyography (EMG) seizure activity. Restimulation was therefore performed 1 min later at 100.9 mC, which induced an EEG seizure lasting 80 s and an EMG seizure lasting 60 s; however, seizure morphology was unstable and postictal suppression was suboptimal. In Session 4, the EEG and EMG seizure durations were 65 and 26 s, respectively, with similarly suboptimal morphology and postictal suppression. The seizure responses in sessions 1 and 4 were considered inadequate, whereas the remaining seven sessions produced adequate seizure responses. The stimulus dose was progressively increased from approximately 152 mC in sessions 2 and 3 to 231.1 mC in Session 4 and 329–332 mC from Session 5 onward. The stimulus train duration was increased from 5.6 to 7.3 s from Session 5.

Beginning around day 111 (after the fourth session), his motivation and emotional expression gradually improved. The symptoms continued to improve, and the patient became able to participate in activities such as muscle training, reading, and interaction with other patients. On day 119 (after six ECT sessions), the MMSE score had improved to 26, while the AS and BPRS‐18 scores decreased to 7 and 26, respectively. Concurrent psychoeducation on substance dependence helped him develop insight into his condition and motivation to maintain abstinence. His general and mental condition stabilized, and at discharge on day 142, the BPRS‐18 score was 25 and the GAF score was 70.

## 3 Discussion

In this case, we first considered the nature of the hallucinations and delusions. During the initial episode, auditory hallucinations resolved, approximately 1 month after cannabis cessation. In the current episode, symptom exacerbation was temporally related to continued daily cannabis use until shortly before admission. However, the psychotic symptoms persisted for approximately 3 months despite antipsychotic treatment. Long‐term cannabis use has been reported to increase the persistence of psychotic symptoms [12], and substance‐induced psychosis may later transition to schizophrenia; registry and meta‐analytic data report transition rates of 27.6% and approximately 34%, respectively, particularly after cannabis‐induced psychosis [6, 13]. Cannabis‐induced psychosis has been reported to more frequently involve expansive mood and ideation, derealization/depersonalization, visual hallucinations, and disturbances of sensorium than acute schizophrenia [14]. In the present case, visual perceptual abnormalities were observed. Reduced responsiveness and an inability to state his date of birth at admission raised the possibility of a mild disturbance of sensorium; however, severe amotivation may also have contributed to these findings. Depersonalization/derealization and expansive mood or ideation were not evident. Although these findings were partly consistent with previously reported features of cannabis‐induced psychosis, they were not diagnostically specific, and clinical differentiation from a primary schizophrenia spectrum disorder remained difficult [15]. The repeated temporal relationship between cannabis use and psychotic symptoms—including resolution of the previous episode approximately 1 month after cannabis cessation and recurrence following resumption of daily cannabis use—supported a substantial contribution of cannabis use, although schizophrenia could not be excluded.

Differentiating persistent amotivation from negative symptoms of schizophrenia is also clinically important [16]. Negative symptoms, including avolition, reduced emotional expression, inactivity, and social withdrawal, overlap substantially with amotivational symptoms [17, 18]. In schizophrenia, negative symptoms are generally categorized as either primary symptoms, which are relatively stable core features of the illness, or secondary symptoms, which may arise from positive symptoms, depression, adverse medication effects, or substance use [19]. In the present case, typical depressive symptoms such as depressed mood and appetite loss were not evident, and the amotivational state persisted despite improvement of positive psychotic symptoms, necessitating consideration of primary negative symptoms. However, the close temporal relationship with cannabis use was notable. Cannabis use in schizophrenia has not been consistently associated with greater negative symptom severity; one meta‐analysis found less severe negative symptoms among recently abstinent cannabis users than nonusers [19]. Evidence for ECT in primary negative symptoms remains inconsistent, with more limited benefit than for positive and general symptoms [20]. Although improvement after ECT in this case does not definitively exclude negative symptoms, the clinical course is difficult to explain solely by primary negative symptoms and suggests the possibility of a reversible cannabis‐related amotivational state.

We also considered the relationship between persistent amotivation and mood disorders. In the ICD‐11, cannabis‐induced mood disorder includes depressed mood but also reduced engagement in pleasurable activities and changes in energy level, making the boundary between this condition and motivation‐related symptoms, as observed in this case, less distinct [9]. However, the ICD‐11 also specifies that symptoms are expected to occur during or shortly after cannabis intoxication or withdrawal, whereas primary mood disorder should be considered when symptoms persist for a substantial period after cessation. Therefore, caution is required when describing this case solely as a cannabis‐induced mood disorder.

Finally, we considered the similarities between the persistent amotivation observed in this case and apathy, as well as the possible mechanisms underlying the effects of ECT. Apathy is characterized by a quantitative reduction in goal‐directed behavior, emotional responsiveness, and indifference [21]. Its clinical presentation overlaps substantially with amotivational syndrome. Treatment generally focuses on the underlying condition and includes environmental interventions, while dopaminergic agents have also been considered [21]. Limited evidence, primarily from patients with acquired brain injury, suggests that amantadine may improve severe apathy or amotivation [22, 23]. Based on these reports, amantadine was initiated as a therapeutic trial. However, amantadine has also been reported to exacerbate psychotic symptoms [24]. Therefore, because psychotic symptoms were still present when amantadine was initiated, it was administered cautiously with close monitoring for worsening hallucinations or delusions. No apparent exacerbation of psychotic symptoms was observed, although improvement in amotivation was limited. Several reports have described improvement in apathy following ECT [25, 26, 27]. Subsequent ECT was associated with marked improvement in motivation, activity, and MMSE performance, with the MMSE score increasing from 19 to 26. However, this change should not necessarily be interpreted as direct evidence of improvement in cognitive function, because MMSE performance may be influenced by attention, effort, motivation, and engagement. Therefore, the improvement in motivation and activity during ECT may have contributed, at least partly, to the higher MMSE score. The biological mechanisms of these conditions should also be considered. Cannabis‐related amotivation and apathy may partly overlap through dysfunction of reward‐related dopaminergic circuits [28, 29, 30, 31]. Chronic cannabis use has been associated with reduced striatal dopamine release [32], whereas ECT may influence dopaminergic neurotransmission. This pathway could be relevant to the improvement observed in this case [33]. However, the mechanism remains speculative, and the improvement may also have reflected spontaneous recovery or the cumulative effects of concomitant treatments.

This study has some limitations. First, this was a single case report, and the efficacy of ECT for persistent amotivation after cannabis‐related psychosis cannot be generalized. Second, although the amotivational state appeared closely related to cannabis use, it was difficult to completely exclude the influence of early schizophrenia spectrum disorder, mood symptoms, medication effects, or cumulative effects of the inpatient environment. Improvement may also have reflected spontaneous recovery or the cumulative effects of concomitant treatments, including antipsychotic therapy, rather than ECT alone. Third, long‐term prognosis was not sufficiently assessed. Considering that substance‐induced psychosis can later transition to schizophrenia spectrum disorders, careful follow‐up remains essential.

In conclusion, this case demonstrates that a persistent amotivational state may remain even after improvement of cannabis‐related psychotic symptoms. Although these symptoms can be conceptualized as cannabis‐related amotivational syndrome, differentiation from the negative symptoms of schizophrenia and mood symptoms remains essential. Clinicians should not automatically regard such symptoms as fixed negative symptoms but should consider the possibility of a potentially reversible cannabis‐related amotivational state. In the present case, ECT may have represented a potential therapeutic option for persistent amotivation. However, because this was a single case and long‐term outcomes remain uncertain, additional cases and longitudinal follow‐up studies are needed to clarify the pathophysiology and optimal treatment strategies for persistent cannabis‐related amotivation.

## Author Contributions

Yukina Kitano, Ryohei Takada, Minobu Ikehara, Kiyori Yamanaka, and Yuka Sunami treated the patient at our hospital. Yukina Kitano, Ryohei Takada, Minobu Ikehara, and Kiyori Yamanaka drafted the manuscript. All authors participated in manuscript discussion, writing, and revision and have read and approved the final version.

## Funding

This research was supported by Japan Agency for Medical Research and Development (AMED) under Grant Number 25dk0307132h0002.

## Consent

Written informed consent was obtained from the patient for the publication of this case report and accompanying clinical information. The consent form is retained by the authors and is available for review by the journal upon request.

## Conflicts of Interest

The authors declare no conflicts of interest.

## Data Availability

Data sharing is not applicable to this article because no datasets were generated or analyzed in the current case report.
